# Comparison of the structure and activity of thio­redoxin 2 and thio­redoxin 1 from *Acinetobacter baumannii*

**DOI:** 10.1107/S2052252523000404

**Published:** 2023-02-08

**Authors:** Ye Ji Chang, Ji Hye Sung, Chang Sup Lee, Jun Hyuck Lee, Hyun Ho Park

**Affiliations:** a College of Pharmacy, Chung-Ang University, Seoul 06974, Republic of Korea; b Department of Global Innovative Drugs, Graduate School of Chung-Ang University, Seoul 06974, Republic of Korea; c College of Pharmacy and Research Institute of Pharmaceutical Science, Gyeongsang National University, Jinju 52828, Republic of Korea; d Unit of Research for Practical Application, Korea Polar Research Institute, Incheon 21990, Republic of Korea; e Department of Polar Sciences, University of Science and Technology, Incheon 21990, Republic of Korea; Chinese Academy of Sciences, China

**Keywords:** *Acinetobacter baumannii*, crystal structure, redox homeostasis, superbugs, thio­redoxin, zinc-finger domains

## Abstract

Thio­redoxin (Trx) is essential in a redox-control system, with many bacteria containing two Trxs: Trx1 and Trx2. Due to a Trx system’s critical function, Trxs are targets for novel antibiotics. Here, a 1.20 Å high-resolution structure of Trx2 from *Acinetobacter baumannii* (abTrx2), an antibiotic resistant pathogenic superbug, is elucidated. By comparing Trx1 and Trx2, it is revealed that the two Trxs possess similar activity, although Trx2 contains an additional N-terminal zinc-finger domain and exhibits more flexible properties in solution. Finally, it is shown that the Trx2 zinc-finger domain might be rotatable and that proper zinc coordination at the zinc-finger domain is critical to abTrx2 activity. This study enhances understanding of the Trx system and will facilitate the design of novel antibiotics.

## Abstract 2

A 1.20 Å high-resolution structure of thio­redoxin 2 (Trx2) from *Acinetobacter baumannii* (abTrx2) is elucidated. Based on comparison of structure and activity with Trx1, it is revealed that the two Trxs in *A. baumannii* possess similar activity, although Trx2 contains an additional N-terminal zinc-finger domain and exhibits more flexible and dynamic properties in solution.

## 1. Introduction

Because oxidative stress damages intracellular macromolecules and affects various cellular processes, maintaining cellular redox homeostasis through proper redox systems is critical for an organism’s survival. Two well known reduction systems that serve as regulators against oxidative stress are the thio­redoxin (Trx) system, which is composed of Trx, thio­redoxin reductase (TrxR) and nicotinamide adenine di­nucleotide phosphate (NADPH); and the glutaredoxin (Grx) system, which is composed of Grx, gluta­thione reductase (GR) and gluta­thione (GSH) (Toledano *et al.*, 2013 ▸; Meyer *et al.*, 2009 ▸). Although the Trx system is found in all living organisms, the Grx system is found in only a few prokaryotic cells (Hirt *et al.*, 2002 ▸; Newton *et al.*, 2009 ▸).

**Figure 1:**
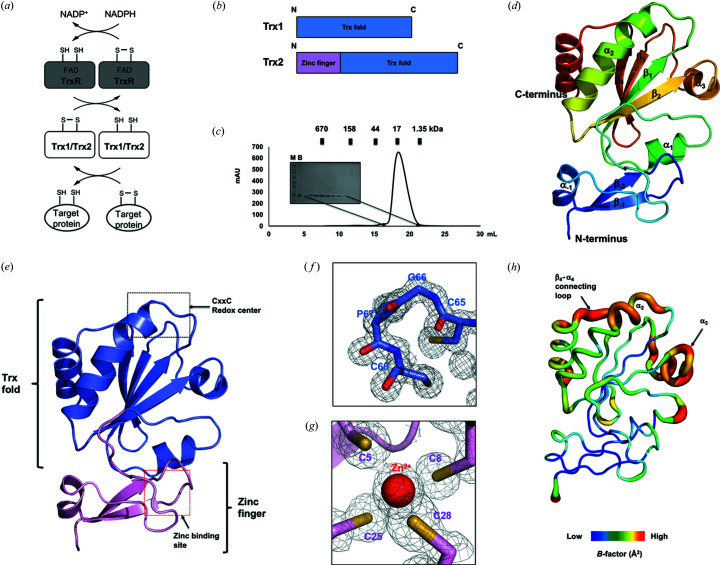
The crystal structure of abTrx2. (*a*) An overview of the function of Trx2 in the Trx redox system of *A. baumannii*. (*b*) Domain composition of Trx2 and Trx1. (*c*) A profile of the SEC performed to purify abTrx2. The eluted positions of standard size markers are shown above the profile. The SDS–PAGE gel loaded with the peak fractions is provided to the left of the main peak. Loaded fractions corresponding to the bands on the SDS–PAGE gel are indicated by black arrows. M and B indicate the size marker and sample before SEC, respectively. (*d*) A cartoon representation of abTrx2. A rainbow color scheme was used for tracing the path from N- to C-terminus. Helices and sheets are labeled with α and β, respectively. (*e*) A cartoon representation showing the domain structure of abTrx2. The black-dotted and red-dotted boxes indicate the CxxC redox center and zinc-binding site that are magnified in panels (*f*) and (*g*), respectively. (*f*) A 2*F*_o_ − *F*_c_ electron-density map contoured at the 1σ level around the CxxC redox center. Residues forming the CxxC redox center are labeled. (*g*) A 2*F*_o_ − *F*_c_ electron-density map contoured at the 1σ level around the zinc-binding site. The cysteine residues involved in zinc coordination are labeled. (*h*) A putty representation of abTrx2 showing the *B*-factor distribution. Rainbow colors range from red to violet to reflect *B*-factor values for *B*-factor visualization. Higher *B*-factor regions are indicated by black arrows.

Trx is a small cytoplasmic enzyme that can reduce di­sulfide bonds in various cellular proteins (Lu & Holmgren, 2014 ▸). Trx has a common CxxC motif (C: cysteine; x: small hydro­phobic residue) in the active site, and this motif is critical to its reductase activity (Collet & Messens, 2010 ▸). To cleave di­sulfide bonds in target proteins, Trx uses the two reduced cysteines at the active site’s CxxC motif. During cleavage, one of the reduced cysteines at the CxxC motif attacks the di­sulfide bond in the target protein. The attacking cysteine then forms a new di­sulfide bond with the other cysteine at the CxxC motif, thereby transferring two electrons to the substrate’s cysteines and cleaving their di­sulfide bond. The oxidized Trx CxxC motif is soon returned to its reduced form by TrxR, which uses a flavin adenine dinucleotide (FAD) cofactor, and cellular NADPH [Fig. 1 ▸(*a*)].

Several bacteria including *Rhodobacter capsulatus*, *Deinococcus radiodurans* and *Escherichia coli* are known to possess two Trxs: Trx1 and Trx2 (Miranda-Vizuete *et al.*, 1997 ▸; Ritz *et al.*, 2000 ▸; Lim *et al.*, 2019 ▸). The most distinct difference between the two Trxs is Trx2’s possession of an additional 30–40 residues at the N-terminus, which forms a zinc-binding motif using four conserved cysteine residues (Kim *et al.*, 2021 ▸; Ye *et al.*, 2007 ▸). Although Trx1 is found in all living cells, Trx2 is absent in some bacteria. In the case of *E. coli*, since Trx2 expression is up-regulated in severe oxidative-stress conditions, whereas Trx1 expression is unchanged, Trx2 might seem to play a supportive role in oxidative-stress conditions, at least in this species (Lundström & Holmgren, 1990 ▸). Due to the Trx system’s essential role in an organism’s survival, the Trx system is considered a promising target for antimicrobial agents. In addition, since Trx is involved in various human diseases, it has been targeted for the treatment of these diseases, such as cancer and rheumatoid arthritis (Benhar *et al.*, 2016 ▸; Zhang *et al.*, 2014 ▸; Liu *et al.*, 2014 ▸; James *et al.*, 2015 ▸; Becker *et al.*, 2001 ▸).

*Acinetobacter baumannii* is one of many drug-resistant superbugs, which are considered one of the biggest threats to public health globally (Kumar, 2016 ▸; Burki, 2018 ▸), and also causes hospital-acquired infections in humans (Antunes *et al.*, 2014 ▸). The importance of developing next-generation antibiotic agents that target superbugs has been highlighted recently, and efforts to do so are ongoing (Wright, 2000 ▸).

*A. baumannii* contains both Trx1 (hereafter abTrx1) and Trx2 (hereafter abTrx2). To gain insight into the structure and activity of Trx2 and compare its properties with those of the recently elucidated structure of Trx1 in *A. baumannii* (Chang & Park, 2022 ▸), we elucidated the high-resolution structure of abTrx2. Using structure analysis and comparisons with structural homologues, mutagenesis studies, and activity assays using the 5,5′-di­thio­bis­(2-nitro­benzoic acid) (DTNB) reduction system, we revealed the unique structural features of abTrx2 and characterized the functional importance of abTrx2’s zinc-finger domain. This study will help deepen our understanding of the two Trxs involved in the redox-controlling system of *A. baumannii.*

## 2. Materials and methods

### 2.1. Protein expression and purification

The full-length *A. baumannii* Trx2 gene (GenBank ID UTY85943.1) was synthesized by Bionics (Seoul, Republic of Korea) and cloned into a pET28a expression vector using NdeI/XhoI restriction sites. The plasmid encoding abTrx2 was transformed into *E. coli* BL21 (DE3) cells. A single colony was selected and cultured for 16 h at 37°C in 5 ml lysogeny broth (LB) containing 50 µg ml^−1^ kanamycin, after which the cells were transferred and cultured in 1 l LB medium. When the optical-density value at 600 nm reached ∼0.7, 0.5 m*M* iso­propyl β-d-1-thiogalactopyran­oside was added to the medium to induce translation of the target gene. The induced cells were further cultured for 18 h at 20°C and harvested by centrifugation at 20°C. The collected cells were resuspended in 40 ml of buffer A (20 m*M* Tris–HCl pH 8.0, 500 m*M* NaCl and 25 m*M* imidazole). The resuspended cells were disrupted by sonication on ice with four bursts of 5 s each and a 25 s interval between two bursts after adding 1 m*M* phenyl­methane­sulfonyl fluoride (Sigma–Aldrich, St Louis, Missouri, USA). The lysed cell suspension was centrifuged at 10 000*g* for 30 min at 4°C to remove the cell debris. The collected supernatant was mixed with nickel nitrilo­tri­acetic acid resin (QIAGEN, Hilden, Germany) through gentle agitation for 2 h at 4°C. The resultant mixture was transferred to a gravity-flow column and washed with 50 ml of buffer B (20 m*M* Tris–HCl pH 8.0, 500 m*M* NaCl and 30 m*M* imidazole). Then, 600 µl of buffer C (20 m*M* Tris–HCl pH 8.0, 500 m*M* NaCl and 250 m*M* imidazole) was loaded onto the column to elute the bound protein. The resulting eluate was subjected to size-exclusion chromatography (SEC) using an ÄKTA Explorer system (GE Healthcare, Chicago, Illinois, USA) equipped with a 24 ml Superdex 200 Increase 10/300 gel-filtration column (GE Healthcare) pre-equilibrated with a SEC buffer (20 m*M* Tris–HCl pH 8.0 and 150 m*M* NaCl). The main peak fractions were collected and dialyzed against a thrombin cleavage buffer (60 m*M* Tris pH 8.0, 300 m*M* NaCl and 1 m*M* CaCl_2_). Afterwards, 50 units of thrombin were used to cleave the N-terminal histidine tag of purified abTrx2 proteins. Cleavage was conducted at 20°C on a shaking incubator for 18 h. The cleaved protein sample was directly applied to the second round of SEC. The main peak fractions were collected and concentrated to 7.3 mg ml^−1^, flash frozen in liquid N_2_, and stored at −80°C until further use. For purification of abTrx1, the same method utilized for purification of abTrx2 was used.

### 2.2. Crystallization and data collection

The hanging-drop vapor-diffusion method was used for crystallization of abTrx2. The protein solution (1 µl) was mixed with an equal volume of the reservoir solution, and the mixed droplet was allowed to equilibrate with 300 µl of the mother liquor in an incubator maintained at 20°C. The initial crystal was produced from a reservoir solution comprising 0.1 *M* HEPES/NaOH pH 7.0 and 15%(*w*/*v*) PEG 20000. The initial crystallization condition was further optimized and high-quality crystals were obtained from a reservoir solution comprising 0.1 *M* HEPES/NaOH pH 7.1 and 14%(*w*/*v*) PEG 20000. The crystal appeared in 28 days and grew to a maximum size of 0.1 × 0.1 × 0.3 mm. For data collection, the crystal was flash cooled in a stream of N_2_ at −178°C in 40% glycerol cryoprotectant. The X-ray diffraction data were collected at the 5C beamline at the Pohang Accelerator Laboratory (Pohang, Republic of Korea). The diffraction data were indexed, integrated and scaled using the *HKL*-2000 program (Otwinowski & Minor, 1997 ▸).

### 2.3. Structure determination and analysis

Protein structure was determined through the molecular replacement (MR) phasing method using the *Phaser* program (McCoy, 2007 ▸) of the *PHENIX* package (Adams *et al.*, 2010 ▸). The previously elucidated *Yersinia pestis* Trx (Protein Data Bank, PDB entry 3p2a; Kim *et al.*, unpublished work), which has 36% amino acid sequence identity with abTrx2, was used as the search model for MR. Model building and refinement were performed using *Coot* (Emsley & Cowtan, 2004 ▸) and *phenix.refine* tools from the *PHENIX* package (Adams *et al.*, 2010 ▸). The quality of the model was validated using *MolProbity* (Chen *et al.*, 2010 ▸). Structural representations were generated using *PyMOL* (DeLano & Lam, 2005 ▸).

### 2.4. Sequence alignment

The amino acid sequences of Trx2 from different species were analyzed using *Clustal Omega* (https://www.ebi.ac.uk/Tools/msa/clustalo/).

### 2.5. SEC multi-angle light-scattering analysis

The absolute molar weight of abTrx2 in solution was determined by multi-angle light scattering (MALS). The target protein was filtered using a 0.2 µm syringe filter and loaded onto a Superdex 200 10/300 gel-filtration column (GE Healthcare) pre-equilibrated in a SEC buffer. The mobile phase buffer was pumped at a rate of 0.4 ml min^−1^ at 25°C. A DAWN TREOS MALS detector (Wyatt Technology, Santa Barbara, California, USA) was connected to the ÄKTA Explorer system (GE Healthcare). The molecular mass of bovine serum albumin was used as the reference value and the absolute molecular mass was assessed using the *ASTRA* program (Wyatt Technology) (Dudka, 2007 ▸).

### 2.6. Mutagenesis of abTrx2

The site-directed mutagenesis was conducted using a QuikChange Mutagenesis Kit (Stratagene) according to the manufacturer’s protocols. Mutagenesis was confirmed by sequencing from Bionics (Seoul, Republic of Korea). Mutant proteins were prepared utilizing the same method used for purification of abTrx2.

### 2.7. abTrx1 and abTrx2 activity analysis by DTNB reduction assay

The DTNB reduction assay was performed to determine the activity of abTrx1, abTrx2 and various abTrx2 mutants. UV radiation was measured using an Implen NanoPhotometer N60 UV–Vis spectrophotometer (Implen, Westlake Village, California, USA). The reaction mixture was prepared by making 1 ml of buffer containing 100 m*M* Tris–HCl buffer (pH 7.0), 10 m*M* ethyl­enedi­amine tetra­acetic acid (EDTA), 1.25 m*M* NADPH, 0.4 µ*M* abTrxR, and 0.5 µ*M* abTrx1 or 0.5 µ*M* abTrx2 or mutants. The reaction was initiated by adding 1.5 m*M* DTNB to the reaction mixture, and the change in optical density at 412 nm was monitored for 15 min at room temperature.

## 3. Results and discussion

### 3.1. Overall structure of abTrx2

Many bacterial species have two Trx proteins, Trx1 and Trx2, for efficient regulation of redox homeostasis in various circumstances. Generally, the sequence identity between Trx1 and Trx2 is ∼30–50%. However, the feature that most distinguishes Trx2 from Trx1 is an additional zinc-finger domain at the N-terminus of Trx2 [Fig. 1 ▸(*b*)]. To compare the structure and activity of Trx2 and Trx1 in *A. baumannii*, and to better understand the Trx system in order to facilitate discovery of novel antibiotics against this species, we performed a structural and biochemical study of abTrx2. For these studies, a recombinant full-length abTrx2 (residues 1–145) was purified by a quick two-step chromatography that was performed through affinity chromatography followed by SEC. In the SEC, abTrx2 was eluted at ∼18.0 ml, where myoglobin (17 kDa size marker) was eluted; we therefore assumed that abTrx2 exists in a monomeric state in solution [Fig. 1 ▸(*c*)].

Initially, the purified recombinant Trx2 protein could not be crystallized. After removing the N-terminal histidine tag with thrombin however, the target protein was successfully crystallized for further structural study. Finally, the 1.20 Å high-resolution crystal structure of abTrx2 was solved with the MR phasing method. The final structural model was refined to *R*_work_ = 17.49% and *R*_free_ = 18.75%. The crystallographic and refinement statistics are presented in Table 1 ▸.

The crystal structure of abTrx2 contains the canonical structural fold of Trx, consisting of a central four-stranded β-sheet (β1–β4) sandwiched by two pairs of α-helices (α1–α4) as well as the zinc-finger domain, consisting of two β-sheets (β1–β2) followed by one α-helix (α1) [Figs. 1 ▸(*d*) and 1 ▸(*e*)]. The CxxC redox center and the zinc-binding site are well formed in the Trx fold and the zinc-finger domain, respectively [Fig. 1 ▸(*e*)]. Interestingly, although abTrx2 was purified and crystallized in an oxidized state, the CxxC redox center of abTrx2 is in a reduced form, characterized by the absence of a di­sulfide-bond between the two cysteine residues at the CxxC motif. Analysis of the electron-density map around the CxxC redox center clearly shows that the electron densities of the two cysteines were disconnected [Fig. 1 ▸(*f*)]. A zinc ion coordinated by four cysteine residues (Cys5, Cys8, Cys25 and Cys28) was detected at the canonical zinc-finger domain [Fig. 1 ▸(*g*)]. *B*-factor analysis indicated that the β4–α4 connecting loop and α3 helix have a higher *B* factor (average 30.29 Å^2^) than other regions (average 16.78 Å^2^) [Fig. 1 ▸(*h*)].

### 3.2. Structure and activity comparison of abTrx2 and abTrx1

**Figure 2:**
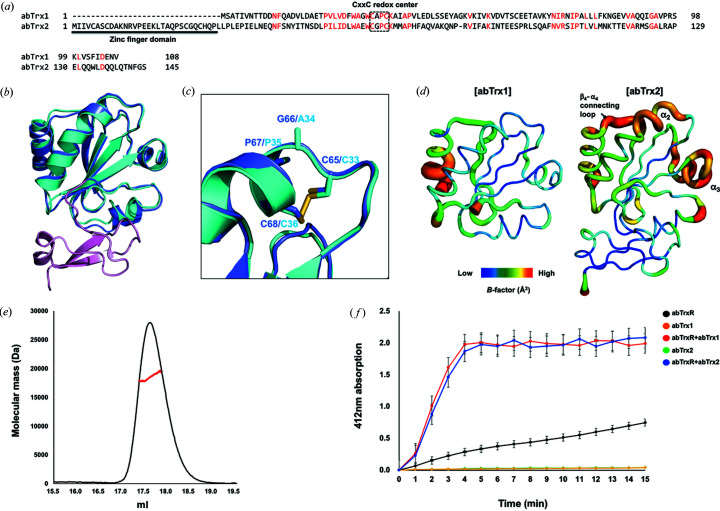
Structure and activity comparison of abTrx2 with abTrx1. (*a*) Sequence alignment of abTrx2 with abTrx1. Conserved residues are marked in red. (*b*) Superimposition of abTrx1 (cyan) and abTrx2 (blue and pink) for structural comparison. (*c*) A magnified view of the CxxC redox centers from the superposed structures of abTrx1 (cyan) and abTrx2 (blue). Residues forming the CxxC redox centers are labeled. (*d*) Comparison of *B*-factor distribution between abTrx1 and abTrx2. Putty representations are provided for visualizing *B*-factor distribution. (*e*) A MALS profile derived from the main SEC peak of abTrx2. The experimental MALS data (red line) are plotted as SEC elution volume (*x* axis) versus absolute molecular mass (*y* axis) distributions on the SEC chromatogram (black) at 280 nm. (*f*) Trx activity analysis by DTNB reduction assay. The reduction of DTNB by abTrxR was tested in the presence of abTrx1 and abTrx2. Error bars indicate the standard deviation for three independent experiments (*n* = 3).

The amino acid sequence identity between abTrx2 and abTrx1 was ∼22% [Fig. 2 ▸(*a*)]. Although the sequence identity is low, the two Trxs structures are nearly identical, having a root-mean-square deviation (RMSD) of 0.8 Å when the two structures of each Trx fold are superposed [Fig. 2 ▸(*b*)]. The additional zinc-finger domain was only detected in the abTrx2 structure [Fig. 2 ▸(*b*)]. The CxxC redox center was sequentially and structurally conserved [Figs. 2 ▸(*a*) and 2 ▸(*b*)]. While the typical sequence of the CxxC motif, including the abTrx2 CxxC motif, is CGPC, the abTrx1 CxxC motif sequence was CAPC [Fig. 2 ▸(*a*)]. Our previous study showed that substituting the abTrx1 alanine residue with glycine or another amino acid containing a similar-sized side chain did not dramatically decrease abTrx1 activity (Chang & Park, 2022 ▸). This result indicated that the presence of alanine or glycine in the CxxC motif is not critical for the functioning of this redox center. The CxxC motif structures of both abTrx1 and abTrx2 are identical, although the abTrx2 motif is in a reduced form, while that of abTrx1 is in an oxidized form. This indicates that oxidization or reduction of the CxxC redox center did not affect the structure of this motif [Fig. 2 ▸(*c*)].

Structural *B*-factor analysis and comparison showed that the abTrx2 α2 helix, where the CxxC redox center was localized, has a relatively higher *B* factor than the abTrx1 α2 helix [Fig. 2 ▸(*d*)]. This indicates that the CxxC motif might be more flexible without the di­sulfide bond between the two cysteines, although the di­sulfide bond did not affect the CxxC motif’s overall structure. Several regions around the abTrx2 CxxC motif, including the β4–α4 connecting loop and α3 helix, also have higher *B* factors than those of abTrx1, indicating that the Trx fold of abTrx2 might be more flexible and dynamic than the Trx fold of abTrx1 [Fig. 2 ▸(*d*)].

Previous study of abTrx1 showed that it exists in a monomeric form in solution. The stoichiometry of the Trx family (*e.g.* whether it exists as a monomer or dimer) is a debated issue (Weichsel *et al.*, 1996 ▸; Campos-Acevedo *et al.*, 2017 ▸). To address this question, we performed a MALS experiment to confirm the stoichiometry of abTrx2 and compare it with that of abTrx1 by determining its absolute molecular weight in solution. The experimentally measured molecular weight of abTrx2 eluted from the main fraction by SEC was 18 490 Da (6.3% fitting error), with a polydispersity value of 1.005 [Fig. 2 ▸(*e*)]. Since the theoretical molecular weight of full-length abTrx2 is 16 308 Da, we concluded that, like abTrx1, abTrx2 exists as a monomer in solution.

Finally, we compared the activity of abTrx2 with that of abTrx1 using a TrxR activity assay, which was based on the reduction of DTNB to 5-thio-2-nitro­benzoic acid (TNB) in the presence of abTrx1 or abTrx2. This assay shows that both abTrx1 and abTrx2 produced a similar amount of TNB that was detected at 412 nm in the presence of abTrxR, indicating that both abTrx1 and abTrx2 possess similar activity [Fig. 2 ▸(*f*)]. Since Trx1 was determined to be more active than Trx2 in an *E. coli* system (Miranda-Vizuete *et al.*, 1997 ▸), the activity difference between Trx1 and Trx2 might be species dependent.

### 3.3. Comparison of the abTrx2 structure with Trx2 structures from different species

**Figure 3:**
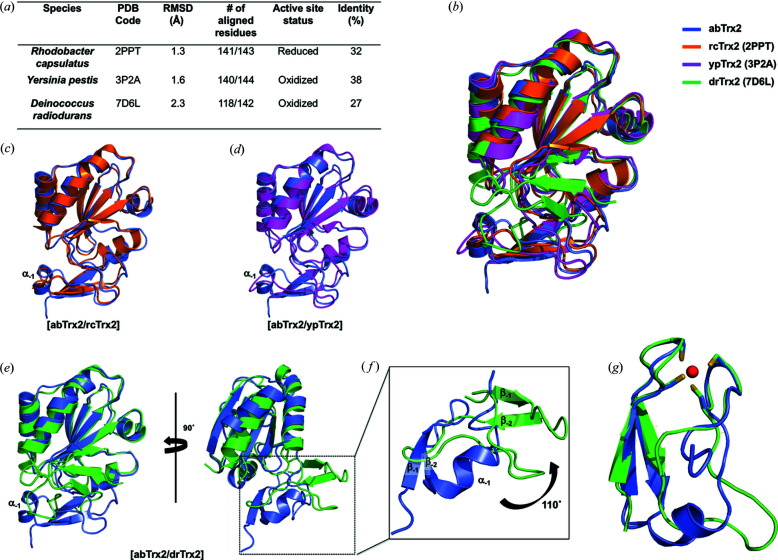
Structural comparison of abTrx2 with isoforms from different species. (*a*) A table summarizing the results of a *DALI* search. (*b*) Structural superimposition of abTrx2 with the top three structures that were identified as structural homologues by the *DALI* server (Holm & Sander, 1995 ▸) (rc: *R. capsulatus*; yp: *Y. pestis*; dr: *D. radiodurans*). The PDB IDs are provided in the parentheses. (*c*),(*d*) Pairwise structural superimposition of abTrx2 (blue) with (*c*) rcTrx2 (orange) and (*d*) ypTrx2 (magenta). (*e*) Pairwise structural comparison of abTrx2 (blue) with drTrx2 (green). (*f*) A magnified view of the zinc-finger domain from the superposed structures of abTrx2 (blue) and drTrx2 (green). (*g*) Structural superposition of the zinc-finger domains of abTrx2 (blue) and drTrx2 (green).

Numerous structural studies of Trx1 have been conducted in the past decades due to the critical function of Trx protein in an organism’s survival (Ingles-Prieto *et al.*, 2013 ▸; Engh *et al.*, 1990 ▸; Goemans *et al.*, 2018 ▸; Starks *et al.*, 2007 ▸; Juniar *et al.*, 2020 ▸; Chang & Park, 2022 ▸). Despite this, only three Trx2 structures, including Trx2 from *R. capsulatus* (rcTrx2, PDB entry 2ppt; Ye *et al.*, 2007 ▸), *Y. pestis* (ypTrx2, PDB entry 3p2a) and *D. radiodurans* (drTrx2, PDB entry 7d6l; Kim *et al.*, 2021 ▸), have been reported [Fig. 3 ▸(*a*)]. Among these three Trx2 structures, the rcTrx2 CxxC redox center was observed in a reduced form, just as that of abTrx2 was. Although Trx2s from different species share low sequence homology (∼27–38% identity), the overall structure of the abTrx2 Trx fold is nearly identical to that of other Trx2s, regardless of whether CxxC motifs are in a reduced or oxidized form. The RMSD value is 1.3 Å in rcTrx2, 1.6 Å in ypTrx2 and 2.3 Å in drTrx2 [Figs. 3 ▸(*b*)–3 ▸(*e*)]. The structure of abTrx2 is most closely related to that of rcTrx2, which has a reduced form of the CxxC motif. Pairwise structural alignments of abTrx2 with the three structural homologues show that only abTrx2 has an α1 helix at the zinc-finger domain [Figs. 3 ▸(*c*)–3 ▸(*e*)]. This part is assigned as a loop in the other homologues. The most distinct structural difference was detected in the comparison of abTrx2 and drTrx2; the zinc-finger domain of drTrx2 is rotated ∼110° compared with that of abTrx2 [Figs. 3 ▸(*e*) and 3 ▸(*f*)]. Although the number of reported Trx2 structures is limited, this structural difference may indicate that the Trx2 zinc-finger domain may be rotatable. Though the function of the zinc-finger domain is still unclear, previous work has indicated that this domain may be involved in target-protein interaction (Ye *et al.*, 2007 ▸). Although the location and overall structure of the zinc-finger domain differ between abTrx2 and drTrx2, the position and structure of the zinc-binding site coordinated by four cysteines are identical [Fig. 3 ▸(*g*)]. This similarity indicates that the strategy for zinc coordination is conserved in the Trx2 family and may be important in zinc-finger domain functioning.

### 3.4. Effect analysis of metal coordination and a conserved Arg13 residue on the activity of abTrx2

**Figure 4:**
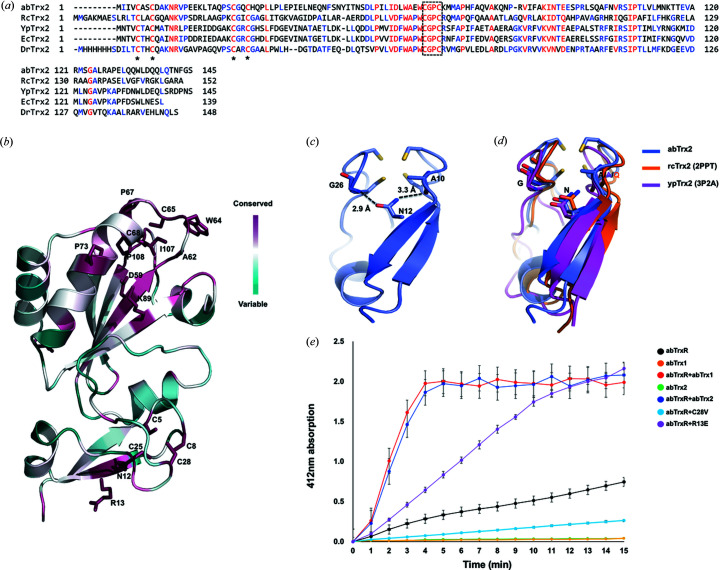
The importance of the zinc-finger domain for abTrx2 activity. (*a*) Sequence alignment of Trx2 from different species (ab: *A. baumannii*; rc: *R. capsulatus*; yp: *Y. pestis*; ec: *E. coli*; dr: *D. radiodurans*). The CxxC motif is enclosed in a black-dotted box. The four cysteine residues involved in zinc ion coordination are indicated by asterisks (*). Completely and partially conserved residues across the different species are shown in red and blue, respectively. (*b*) A graphic representation of abTrx2 colored according to the degree of amino acid sequence conservation generated by the *ConSurf* server. (*c*) A cartoon figure showing the hydrogen bonds mediated by Asn12 in the zinc-finger fold of abTrx2. (*d*) A cartoon figure showing the hydrogen bonds mediated by conserved Asn12 (Asn22 of rcTrx2 and Asn12 of ypTrx2) in the zinc-finger fold. (*e*) abTrx2 mutant activity analysis by DTNB reduction assay. The reduction of DTNB by abTrxR was tested in the presence of abTrx2 mutants. Error bars indicate the standard deviation for three independent experiments (*n* = 3).

Phylogenetic analysis of homologous Trx2 sequences from 500 species using the *ConSurf* server indicated that the residues that form the CxxC motif and the core of the Trx fold are completely conserved [Figs. 4 ▸(*a*) and 4 ▸(*b*)] (Ashkenazy *et al.*, 2016 ▸). In the case of the zinc-finger domain, the four cysteine residues forming a zinc-binding site were completely conserved. In addition to these cysteine residues, Asn12 and Arg13 residues on the zinc-finger domain were completely conserved across the considered species, except the species of rcTrx2 that contains a lysine residue instead of Arg13 [Fig. 4 ▸(*a*)]. Since Asn12 was involved in structural maintenance by locating the inside of the zinc-finger fold, it is expected that Asn12 is conserved because of its role in maintaining a proper 3D structure. Indeed, the structural analysis indicated that Asn12 of abTrx2 formed hydrogen bonds with the main chains of Ala10 and Gly26 [Fig. 4 ▸(*c*)]. The importance of the Asn12 residue for maintaining the proper zinc-finger fold was conserved across the species by forming similar hydrogen-bond networks [Fig. 4 ▸(*d*)]. However, the reason for the preservation of the surface residue Arg13 was hard to predict. Since conserved residues could be critical to the activity of Trx2, we tested the effect of an R13E mutant on the activity of abTrx2. We also analyzed the effect of the coordinated zinc ion in the zinc-finger domain on the activity of abTrx2. To test this, we mutated the Cys28 residue to valine in order to generate a C28V mutant that lacks a zinc ion in the zinc-finger domain. According to the DTNB reduction assay, abTrxR showed strong TrxR activity in the presence of purified abTrx1 or abTrx2. However, the abTrx2 C28V variant completely lost its TrxR activity, while TrxR in the R13E variant decreased by almost half [Fig. 4 ▸(*e*)]. These results indicate that proper zinc coordination is critical to the activity of abTrx2 and that surface-exposed Arg13 affects abTrx2 activity.

In conclusion, a high-resolution structure of Trx2 from a drug-resistant superbug, *A. baumannii*, was provided in this study. It was shown that abTrx2 exists as a monomer in solution and contains an additional zinc-finger domain at the N-terminus that is absent in abTrx1. Although the overall structure of the Trx fold of abTrx2 is nearly identical to that of abTrx1, *B*-factor analysis indicated that abTrx2 might be more flexible and dynamic in solution. The activity of abTrx1 and abTrx2 was almost identical. Compared with Trx2 homologues from different species, abTrx2 contains a unique additional helix (α1) at the zinc-finger domain. By comparing the structure of abTrx2 with that of drTrx2, we identified that the Trx2 zinc-finger domain might be rotatable. Finally, we revealed that proper zinc coordination at the zinc-finger domain is critical to the activity of abTrx2. In addition, we showed that the conserved surface-exposed Arg13 residue on the zinc-finger domain affects abTrx2 activity. Because the Trx system is involved in various processes integral to an organism’s survival, such as cellular redox homeostasis and cell growth and differentiation, the protein components in the Trx system, including Trx2, are considered to be a prospective target for antibiotic development. The current study has provided information on the structure and activity of abTrx2 that may help in the specific drug design for targeting the Trx system of *A. baumannii*.

## Supplementary Material

PDB reference: Trx2 from *Acinetobacter baumannii*, 7ysi

## Figures and Tables

**Table 1:** Data-collection and refinement statistics Values for the outermost resolution shell are in parentheses.

Data collection	
Space group	*P*2_1_2_1_2_1_
Unit-cell parameter	
*a*, *b*, *c* (Å)	34.59, 55.01, 78.09
α, β, γ (°)	90, 90, 90
Resolution range (Å)	27.42–1.20
Total reflections	580954 (41483)
Unique reflections	45597 (4106)
Multiplicity	12.7 (10.1)
Completeness (%)	96.64 (92.70)
〈*I*/σ(*I*)〉	15.42 (1.50)
*R*_merge_ (%)†	9.36 (24.35)
Wilson *B* factor (Å^2^)	14.86
	
Refinement	
Resolution range (Å)	27.42–1.20
Reflections	45582 (4105)
*R*_work_ (%)	17.49 (47.86)
*R*_free_ (%)	18.75 (41.77)
No. of molecules in the asymmetric unit (ASU)	1
No. of non-hydrogen atoms	1341
Macromolecules	1122
Solvent	217
Average *B*-factor values (Å^2^)	18.42
Macromolecules	16.78
Solvent	26.92
Ramachandran plot	
Favored/allowed/outliers (%)	98.58/1.42/0.00
Rotamer outliers (%)	0.00
Clashscore	2.23
RMSD bonds (Å)/angles (°)	0.009/1.007

† *R*_merge_ = 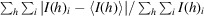 , where *I*(*h*) is the observed intensity of reflection *h* and 〈*I*(*h*)〉 is the average intensity obtained from multiple measurements.
